# Set stability and synchronization of generalized asynchronous probabilistic Boolean networks with impulsive effects

**DOI:** 10.1371/journal.pone.0318038

**Published:** 2025-02-12

**Authors:** Fukun Liu, Yujie Sun, Chuan Zhang, Lunshi Xu, Hao Zhang

**Affiliations:** 1 College of Computer Science, Taiyuan University of Technology, Jinzhong, China; 2 China Northern Vehicle Research Institute, Beijing, China; 3 China North Artificial Intelligence & Innovation Research Institute, Beijing, China; 4 School of Mathematical Science, Qufu Normal University, Qufu, China; University of Milano–Bicocca: Universita degli Studi di Milano-Bicocca, ITALY

## Abstract

This paper investigates the set stability of generalized asynchronous probabilistic Boolean networks (GAPBNs) with impulsive effects. To this end, an efficient algorithm is designed to determine the largest invariant set of a given set. A necessary and sufficient criterion is then derived to determine set stability of GAPBNs with impulsive effects. Subsequently, the global stability and synchronization of GAPBNs with impulsive effects were verified by selecting different sets. Finally, examples are given to illustrate the results on set stability and synchronization.

## Introduction

Recently, with the development of systems biology, gene regulatory networks (GRNs) research has received increasing attention. GRNs refer to networks formed by interactions between genes located in a cell or a genome. Especially, Boolean networks (BNs) were first proposed by Kaufman (1969) to describe GRNs [1]. For BNs, each node has only two states, “0” and “1”, corresponding to the state “on” or “off” of each gene. Especially, “on” indicates the gene expression, while “off” indicates no expression. What’s more, the interaction between two genes is depicted by the Boolean logic on the edge.

In previous studies, it is difficult to process logical relationships in BNs. In recent years, Cheng et al. propose a new tool named “semi-tensor product (STP)” [2] to effectively convert logical expressions into algebraic forms. These algebraic forms can be further analyzed with classical mathematical methods. Since then, lots of researches have emerged on BNs, such as stability [3], controllability [4, 5], synchronization [6], observability [7], robustness [8], identification [9] and output tracking [10]. Meanwhile, STP has also been applied to researches related to game theory [11, 12].

However, most of existing researches were conducted on BNs with synchronous updates. In actually, in the realm of biological systems, such as gene regulatory networks, the asynchronous nature of molecular interactions is a fundamental characteristic. Unlike traditional synchronous models, asynchronous Boolean networks can capture the fact that different genes activate, inhibit, or change states at varying timescales. This asynchronous behavior is crucial as it reflects the real-time dynamics within cells more accurately, allowing for a better understanding of complex biological processes such as cell differentiation and disease progression. Harvey (1997) proposed an asynchronous random Boolean networks (ARBNs), in which only one node is randomly selected for update at each time step. Further researches have focused on attractors [13] and dynamics [14] of ARBNs. Greil et al. (2007) proposed deterministic asynchronous Boolean networks (DABNs), in which the update rules are fixed [15]. Subsequently, Zhang et al. investigated the synchronization of DABNs [16]. Carlos et al. proposed generalized asynchronous Boolean networks (GABNs) [17], in which, unlike ARBNs, multiple nodes could be simultaneously updated. For GABNs, the attractors [18] and controllability [19] has been further examined. Zhang et al. proposed the model of asynchronous probabilistic Boolean networks (APBNs) [20] and studied the controllability of delayed generalized asynchronous Boolean control networks under disturbances [21]. Tong et al. studied the fault detectability of asynchronous delay Boolean control networks using data control [22]. There is a large amount of inherent randomness in biological systems, such as random fluctuations in gene transcription and translation processes, uncertainty in intermolecular interactions. The probability characteristics in GAPBNs can simulate this randomness. However, less focus has been paid on GAPBNs. Based on the above discussion, the study of GAPBNs is meaningful.

During the process of network evolution, external interference is often encountered, and pulse effects are a common type of interference. Therefore, it is necessary to consider pulse effects in the construction of BNs. Li et al. proposed the model of BNs with impulsive effects [23]. There have been many researches on synchronous BNs with impulsive effects, including synchronization of swithed Boolean networks (SBNs) [24], stability of stochastic SBNs [25] and set stabilization of Boolean control networks (BCNs) [26]. Therefore, we can conduct research on GAPBNs with impulsive effects.

Considering that the constructed network can be stable into a set of states, it is essential for BNs to investigate set stability and stabilization. To this end, the largest invariant set, in some existing studies, has been used to investigate the set stability for BNs [27] and SBNs [28, 29]. Besides, the largest control invariant set has been used to investigate the set stabilization for BCNs [27], BCNs with impulsive effects [26] and BCNs with state dependent random impulses [30]. In addition, in recent years, there have been many studies on the set stability and set stabilization of BNs and BCNs [31–33]. However, few researches has been conducted on the set stability of GAPBNs with impulsive effects.

This study aims to investigate the set stability and synchronization of GARBNs with impulsive effects. The main contributions fo the study are listed as below:

(1) Design an algorithm to determine the largest invariant set of a given set for GAPBNs with impulsive effects;
(2) Propose a sufficient and necessary condition for the set stability of GAPBNs with impulsive effects;
(3) Derive criteria for determining global stability and internal synchronization by testing on the sets with different states.

The rest paper is organized as follows: Section 2 briefly describes STP, and proposes the GAPBNs model with impulsive effects. In Section 3, a theorem and corollaries about set stability, including global stability and synchronization, are given for GAPBNs with impulsive effects. Besides, some examples are provided in Section 4. Finally, Section 5 concludes for the results in this study.

## Materials and methods

### STP of matrices

The necessary symbols and concepts are introduced below. For details, please refer to [27, 34].

(1) $\Delta_{n}≔\{\delta_{n}^{r},r=1,2,\cdots n\}$, where $\delta_{n}^{r}$ repersents the *r*-th column of *n*-dimensional identity matrix *I*_*n*_;
(2) For convenience, define $\left[\delta_{n}^{r_{1}},\delta_{n}^{r_{2}},\cdots ,\delta_{n}^{r_{m}}\right]$ as *δ*_*n*_[*r*_1_, *r*_2_, ⋯, *r*_*m*_] and $1_{n}≔{\underset{︸}{{\left(1,1,\ldots ,1\right)}^{T}}}_{n}$;
(3) For two matrices $A\in R^{m\times n}$ and $B\in R^{p\times q}$, the STP of *A* and *B* is
  $$\begin{array}{c} A⋉B=\left(A⊗I_{\frac{t}{n}}\right)\left(B⊗I_{\frac{t}{p}}\right), \end{array}$$
  where ⊗ is used for the tensor product, and *t* is the least common multiple of *n* and *p*;
(4) If $A\in R^{m\times n}$,
  $$\begin{array}{c} Col\left(A\right)≔\{Col_{i}\left(A\right)|i=1,2,\cdots ,n\}, \end{array}$$
  $$\begin{array}{c} Row\left(A\right)≔\{Row_{i}\left(A\right),i=1,2,\cdots ,m\}, \end{array}$$
  where *Col*_*i*_(*A*) is the *i*-th column of matrix *A* and *Row*_*i*_(*A*) is the *i*-th row of matrix *A*;
(5) Let *f*: *D*^*n*^ → *D* be a Boolean mapping, there exists a unique structure matrix $M_{f}\in L_{2\times 2^{n}}$ for *f* such that
  $$\begin{array}{c} f\left(x_{1},x_{2},\cdots ,x_{n}\right)=M_{f}{⋉}_{i=1}^{n}x_{i},x_{i}\in \Delta_{2} \end{array}$$
  where ${⋉}_{i=1}^{n}x_{i}=x_{1}⋉\cdots ⋉x_{n}$;
(6) For two matrices $A\in R^{m\times r}$ and $B\in R^{p\times r}$, the Khatri-Rao product of *A* and *B* is defined as
  $$\begin{array}{c} A*B=[Col_{1}\left(A\right)⋉Col_{1}\left(B\right),Col_{2}\left(A\right)⋉Col_{2}\left(B\right),\cdots ,Col_{r}\left(A\right)⋉Col_{r}\left(B\right)]\in M_{mp\times r}. \end{array}$$

### Model and algebraic representation

The *n*-node GAPBN with impulsive effects can be depicted as follows:

$$\begin{array}{c} x_{i}\left(t+1\right)=\{\begin{array}{c} f_{i}(x_{1}\left(t\right),x_{2}\left(t\right),\cdots ,x_{n}\left(t\right)),\;\text{updated}\;\text{and}\;t\neq t_{k}-1,\\ x_{i}\left(t\right),\;\text{not}\;\text{updated}\;\text{and}\;t\neq t_{k}-1,\\ e_{i}(x_{1}\left(t\right),x_{2}\left(t\right),\cdots ,x_{n}\left(t\right)),\;t=t_{k}-1, \end{array} \end{array}$$ (1)

where $\{t_{k}|k\in Z_{+}\}\subseteq Z_{+}$, satisfying 0 < *t*_0_ < *t*_1_ < *t*_2_ < ⋯ < *t*_*k*_ < ⋯, is the impulsive time sequence; *x*_*i*_ ∈ Δ_2_, *i* = 1, 2, ⋯, *n* is the state variable of the *i*-th node; *f*_*i*_: *D*^*n*^ → *D* and *e*_*i*_: *D*^*n*^ → *D* are logical functions.

The probability of updating at each node is represented by *p*_*i*,*j*_, where *i* ∈ {1, 2, ⋯, *n*} represents the node, *j* ∈ {1, 2} represents the update status and *p*_*i*,1_ + *p*_*i*,2_ = 1. *j* = 1 represents node status updates, while *j* = 2 does not. For instance, *p*_2,1_ = 0.5 indicates that the probability of the second node updating is 0.5.

With STP tool, the GAPBN model with impulsive effects can be transformed into the following linear algebraic form.

$$\begin{array}{c} x\left(t+1\right)=\{\begin{array}{c} F_{m}x\left(t\right),\;t\neq t_{k}-1,\;m\in \{0\cdots 2^{n}-1\},\\ Ex\left(t\right),\;t=t_{k}-1, \end{array} \end{array}$$ (2)

where *F*_*m*_ = *M*_1,m_*M_2,m_*⋯*M_*m*,*n*_ is the structure matrix for asynchronous update, and $E=M_{e_{1}}*M_{e_{2}}*\ldots *M_{e_{n}}$ is the structure matrix under impulsive effects. $x\left(t\right)={⋉}_{i=1}^{n}x_{i}$. *M*_*i*,*j*_ represents the state transition matrix of the *i*-th node under the *j*-th asynchronous update. *m* represents the asynchronous update situation.

Let *F* = [*F*_0_, ⋯, *F*_2^*n*^−1_], $P={\left[P_{0},\ldots ,P_{2^{n}-1}\right]}^{T}$, and *P*_*i*_ = *P*_*r*_{*F*_*m*_ = *F*_*i*_}, where *P*_*i*_ = *p*_1,*j*_ × *p*_2,*j*_ × ⋯ × *p*_*n*,*j*_. For instance, *P*_1_ = *p*_1,1_ × *p*_2,2_ × ⋯ × *p*_*n*,2_ represents the probability that only the first node will be updated. Especially, *P*_0_ indicates no node updated. Let *G*_1_ = *F* ⋉ *P* and *G*_2_ = *E*, the overall Boolean network expected value of *x*(*t* + 1) can be expressed as

$$\begin{array}{c} Ex\left(t+1\right)=\{\begin{array}{c} G_{1}Ex\left(t\right),\;t\neq t_{k}-1,\\ G_{2}Ex\left(t\right),\;t=t_{k}-1. \end{array} \end{array}$$ (3)

**Remark 1.** The GAPBNs with impulsive effects model is constructed. Compared to ARPN [13], DABNs [15] and APBNs [20], GAPBNs have a more universal generalized asynchronous mechanism. Besides, GAPBNs with impulsive effects, with the addition of pulse effects and probabilities, are more realistic than GABNs [21, 22]. These complex conditions can better reflect the real-world complexity in the relevant systems. Moreover, the probability of GAPBNs is reflected in the update mechanism, while the probability of probabilistic Boolean networks is reflected in the selection of logical functions.

## Results and discussion

This section investigates the set stability and synchronization of GAPBNs with impulsive effects. The relevant theorems are put forward and proved. First of all, set stability is defined for GAPBNs with impulsive effects.

**Definition 1.** Given an invariant set $\Omega \subseteq \{\delta_{2^{n}}^{i_{1}},\cdots ,\delta_{2^{n}}^{i_{r}}\}$. GAPBN with impulsive effects (1) is said to be finite-time Ω-stable with probability one, if there exists a positive integer *k* such that

$$\begin{array}{c} P_{r}\{x(t;x(0))\in \Omega \}=1, \end{array}$$

holds for any integer *t* ≥ *k* and any *x*(0) ∈ *D*^*n*^. Here, *x*(*t*; *x*_0_) represents the state trajectory at time *t* and is initially set to *x*_0_.

### Invariant subset

To deal with the set stability problem of BNs for a given state set Ω, conventional approaches, as reported in [27], calculate the invariant subset, which is defined as below.

**Definition 2.** A set Ω ⊆ Δ_2^*n*^_ is called an invariant set of GAPBN with impulsive effects (1), if *P*(*x*(*t*; *x*_0_) ∈ Ω∣*x*_0_ ∈ Ω) = 1 is ture for any time *t*.

Particularly, the largest invariant set, denoted by *I*(Ω), is the invariant set that contains the largest number of states among all invariant sets.

However, these conventional methods are not competent for GAPBNs with impulsive effects, the occurrence of impulses may cause the state to deviate from the largest invariant set. Lin (2020) used stricter conditions to determine the largest control invariant set for synchronous BCNs with impulse effects [26]. In our study, this method is improved to decide the largest invariant set for GAPBNs with impulsive effects, as described in Algorithm 1.

**Algorithm 1** Algorithm for the Largest Invariant Set

1: **Initialize**: Ω[*t*] ≔ ∅, *t* ≔ 0, *I*(Ω)≔ Ω, Ω[0] ≔ Ω.

2: **while** |*I*(Ω)| > 0 **do**

3:  *t* = *t* + 1.

4:  **for** each state $\delta_{2^{n}}^{i}\in \Omega \left[t-1\right]$ **do**

5:   **if** *t* ≠ *t*_*k*_ **then**

6:    $\psi ≔\{\delta_{2^{n}}^{i}:Col_{i}\left(\sum_{j\in I(\Omega (t-1))}Row_{j}\left(G_{1}\right)\right)\neq 1\}$

7:    Ω(*t*) ≔ Ω(*t* − 1)\*ψ*

8:   **else**

9:    $\zeta ≔\{\delta_{2^{n}}^{i}:Col_{i}\left(G_{2}\right)\notin \Omega \left[t-1\right]\}$

10:    Ω(*t*)≔ Ω[*t* − 1]\*ζ*

11:   **end if**

12:  **end for**

13:  **if** Ω(*t*) = Ω(*t* − 1) = Ω(*t* − 2) with *t* = *t*_*k*_, *t* − 1 ≠ *t*_*k*_ **then**

14:   **return** *I*(Ω)≔ Ω[*t*]

15:  **end if**

16: **end while**

**Theorem 1.** Considering the system (3), for a given set Ω, the largest invariant set *I*(Ω) of Ω can be obtained by **Algorithm 1**.

**Proof.** Firstly, verify that the *I*(Ω) generated by Algorithm 1 is an invariant set. Assuming that $Ex\left(t\right)=\delta_{2^{n}}^{i}\in I\left(\Omega \right)\subseteq \Omega$, one can get

(1) if *t* + 1 = *t*_*k*_, then $Ex\left(t+1\right)=G_{2}Ex\left(t\right)=Col_{i}\left(G_{2}\right)$;
(2) otherwise, $Ex\left(t+1\right)=G_{1}Ex\left(t\right)\in I\left(\Omega \right)$.

Hence, Ex(t+1)∈I(Ω) and *I*(Ω) is a invariant set of Ω.

Next, prove that *I*(Ω) is the largest invariant set. Assuming that there is an invariant set Ω′ satisfying $I\left(\Omega \right)⫋\Omega^{\prime }\subseteq \Omega$ and there exists a $x\left(t\right)=\delta_{2^{n}}^{ℵ}\in \Omega^{\prime }\backslash I\left(\Omega \right)$, base on the Definition 2, it yields $Ex\left(t+l\right)\in \Omega^{\prime }$, $\forall l\in N_{+}$. Thus, there exist three time steps *t* + *l*_1_ = *t*_*k*_, *t* + *l*_1_ − 1 ≠ *t*_*k*_, *t* + *l*_1_ − 2 such that Ω[*t* + *l*_1_] = Ω[*t* + *l*_1_ − 1] = Ω[*t* + *l*_1_ − 2]. On the basis of Algorithm 1, one can obtain that $\delta_{2^{n}}^{ℵ}\in I\left(\Omega \right)$. Hence, the largest invariant set is *I*(Ω).

**Remark 2.** The determination of the largest invariant set in this article is different from the traditional [27]. Because a network reaches set stability without the influence of pulses, and at the pulse moment, the state may jump out of the original largest invariant set and reach any state. The constraint conditions for determining the largest invariant set of Boolean control networks affected by pulses under synchronous updates are given in [26], ensuring that they do not leave the determined largest invariant set even at the pulse moment. However, the previous methods mainly focused on synchronous updates, while asynchronous updates also need to be considered. This article proposes a method for determining the largest invariant set of Boolean networks affected by pulses under asynchronous updates, based on the above methods.

### Set stability

After determining the largest invariant set *I*(Ω), the set stability of GAPBN with impulsive effects (1) can be further investigated.

**Lemma 1.** [27]: Given an invariant subset Ω ⊆ Δ_2^*n*^_, BN is Ω-stable if and only if it is *I*(Ω)-stable.

According to Lemma 1, the concept of finite-time Ω-stable can be defined as follows.

**Definition 3.** GAPBN with impulsive effects (1) is said to be finite-time Ω-stable with probability one, if there exists a positive integer *k* such that

$$\begin{array}{c} P_{r}\{x\left(t;x\left(0\right)\right)\in I(\Omega )\}=1 \end{array}$$

holds for any integer *t* ≥ *k* and any *x*(0) ∈ *D*^*n*^.

For the algebraic form (3), given *x*(0) ∈ Δ_2^*n*^_, by a simple iteration, one can see that

$$\begin{array}{c} \begin{array}{cc} & Ex\left(1\right)=F_{1}x\left(0\right)≔{\hat{F}}_{1}x\left(0\right),\\ & Ex\left(2\right)=G_{1}Ex\left(1\right)=G_{1}{\hat{F}}_{1}x\left(0\right)≔{\hat{F}}_{2}x\left(0\right),\\ & \;\;\vdots \\ & Ex\left(t_{1}-1\right)=G_{1}Ex\left(t_{1}-2\right)=G_{1}{\hat{F}}_{t_{1}-2}x\left(0\right)≔{\hat{F}}_{t_{1}-1}x\left(0\right),\\ & Ex\left(t_{1}\right)=G_{2}Ex\left(t_{1}-1\right)=G_{2}{\hat{F}}_{t_{1}-1}x\left(0\right)≔{\hat{F}}_{t_{1}}x\left(0\right),\\ & \;\;\vdots \end{array} \end{array}$$ (4)

For given positive integer *k*, perform the above iteration, one can finally get $Ex\left(k\right)={\hat{F}}_{k}x\left(0\right)$, where

$$\begin{array}{c} {\hat{F}}_{k}=\{\begin{array}{c} F_{1},\;\text{when}\;k=1,\\ G_{1}{\hat{F}}_{k-1},\;\text{when}\;k\neq t_{k},\\ G_{2}{\hat{F}}_{k-1},\;\text{when}\;k=t_{k}. \end{array} \end{array}$$ (5)

Based on the result and Definition 3, one has the following theorem.

**Theorem 2.** Given a subset Ω ⊆ Δ_2^*n*^_, GAPBN with impulsive effects (1) is finite-time Ω-stable with probability one, if and only if there exists a positive integer *k* such that

$$\begin{array}{c} \sum_{j\in I(\Omega )}\;Row_{j}\left({\hat{F}}_{k}\right)=1_{2^{n}}^{T}. \end{array}$$ (6)

**Proof.** (Sufficiency): Assuming that (6) holds. Prove that Ex(t;x(0))∈I(Ω) holds for any integer *t* ≥ *k* and any *x*(0) ∈ Δ_2^*n*^_, where $I\left(\Omega \right)=\{\delta_{2^{n}}^{j_{1}},\cdots ,\delta_{2^{n}}^{j_{*}},\cdots ,\delta_{2^{n}}^{j_{r}}\}$, *r* is the element number of *I*(Ω).

Since

$$\begin{array}{c} \sum_{j\in I(\Omega )}\;Row_{j}\left({\hat{F}}_{k}\right)=1_{2^{n}}^{T}, \end{array}$$

one can see that $Ex\left(k;x\left(0\right)\right)={\hat{F}}_{k}x\left(0\right)=\delta_{2^{n}}^{j_{*}}\in I\left(\Omega \right)$ holds for any *x*(0) ∈ Δ_2^*n*^_.

Assuming that $Ex\left(\xi -1;x\left(0\right)\right)=\delta_{2^{n}}^{j_{1}}\in \Omega$ holds for any *x*(0) ∈ Δ_2^*n*^_, where *ξ* − 1 > *k*. Now, we prove the case of *t* = *ξ*.

- if *ξ* ≠ *t*_*k*_, then
  $$\begin{array}{c} Ex\left(\xi ;x\left(0\right)\right)=\sum_{m=0}^{2^{n}-1}\;P_{m}F_{m}Ex\left(\xi -1;x\left(0\right)\right)=G_{1}\delta_{2^{n}}^{j_{1}}=\delta_{2^{n}}^{j_{*}}\in I\left(\Omega \right). \end{array}$$
- if *ξ* = *t*_*k*_, one has
  $$\begin{array}{c} Ex\left(\xi ;x\left(0\right)\right)=E\cdot Ex\left(\xi -1;x\left(0\right)\right)=G_{2}\delta_{2^{n}}^{j_{1}}=\delta_{2^{n}}^{j_{*}}\in I\left(\Omega \right). \end{array}$$

Thus, one can conclude that Ex(t;x(0))∈I(Ω) holds for any integer *t* ≥ *k*, any *x*(0) ∈ Δ_2^*n*^_. Therefore, there exists a positive integer *k* such that

$$\begin{array}{c} P_{r}\{x(t;x(0))\in I(\Omega )\}=1 \end{array}$$

holds for any integer *t* ≥ *k* and any *x*(0) ∈ Δ_2^*n*^_. By Definition 3, GAPBN with impulsive effects (1) is finite-time Ω-stable with probability one.

(Necessity): Supposing that GAPBN with impulsive effects (1) is finite-time Ω-stable with probability one. Then, from Definition 3, there exists a positive integer *k* such that

$$\begin{array}{c} P_{r}\{x(k;x(0))\in I(\Omega )\}=1 \end{array}$$

holds for any initial state *x*(0) ∈ Δ_2^*n*^_. Hence, Ex(k;x(0))∈I(Ω). From (5), one has ${\hat{F}}_{k}x\left(0\right)\in I\left(\Omega \right)$, which along with the arbitrariness of *x*(0) shows that

$$\begin{array}{c} \sum_{j\in I(\Omega )}\;Row_{j}\left({\hat{F}}_{k}\right)=1_{2^{n}}^{T}. \end{array}$$

### Stability and synchronization

The definitions of stability and synchronization are given as follows.

**Definition 4.** A state *x*_*d*_ ∈ Δ_2^*n*^_ of the GAPBN with impulsive effects is said to be finite-time stable at *x*_*d*_ with probability one, if there exists an positive integer *k* such that

$$\begin{array}{c} P_{r}\{x\left(t;x\left(0\right)\right)=x_{d}\}=1,\;\forall t\geq k,\forall x_{0}\in \Delta_{2^{n}}. \end{array}$$ (7)

**Definition 5.** The GAPBN with impulsive effects (1) is said to be inner synchroized with probability one, if there, for any initial node state *x*_*i*_(0)∈Δ_2_, is a positive integer *k* such that

$$\begin{array}{c} P_{r}\{x_{1}\left(t\right)=x_{2}\left(t\right)=\cdots =x_{n}\left(t\right)\}=1, \end{array}$$

where *t* ≥ *k*, and *i* = 1, 2, ⋯, *n*.

Similar to the iterative process in (4), for an any given positive integer *k*, we have $Ex\left(k\right)={\tilde{F}}_{k}x\left(0\right)$, where

$$\begin{array}{c} {\tilde{F}}_{k}=\{\begin{array}{c} F_{1},\;\text{when}\;k=1,\\ G_{1}{\tilde{F}}_{k-1},\;\text{when}\;k\neq t_{k},\\ G_{2}{\tilde{F}}_{k-1},\;\text{when}\;k=t_{k}. \end{array} \end{array}$$ (8)

Based on (8) and Definition 4, one has the following corollary.

**Corollary 1.** Given an equilibrium *x*_*d*_. Assuming that *G*_*i*_ *x*_*d*_ = *x*_*d*_, *i* = 1, 2. Then, GAPBN with impulsive effects (1) is finite-time stable at *x*_*d*_ with probability one, if and only if there exists a positive integer *k* such that

$$\begin{array}{c} Row_{d}\{{\tilde{F}}_{k}\}=1_{2^{n}}^{T}. \end{array}$$ (9)

**Proof.** (Sufficiency): Assuming that (9) holds. We prove that $Ex\left(t;x\left(0\right)\right)=\delta_{2^{n}}^{\chi }$ holds for any integer *t* ≥ *k*, any *x*(0)∈Δ_2^*n*^_.

Since $Row_{d}\{{\tilde{F}}_{k}\}=1_{2^{n}}^{T}$, one can see that $Ex\left(k;x\left(0\right)\right)={\tilde{F}}_{k}x\left(0\right)=\delta_{2^{n}}^{\chi }$ holds for any *x*(0) ∈ Δ_2^*n*^_.

Assuming that $Ex\left(\mu -1;x\left(0\right)\right)=\delta_{2^{n}}^{\chi }$ holds for any *x*(0) ∈ Δ_2^*n*^_, where *ξ* − 1 > *k*. Now, we prove the case of *t* = *ξ*.

- if *ξ* ≠ *t*_*k*_, then
  $$\begin{array}{c} Ex\left(\xi ;x\left(0\right)\right)=\sum_{m=0}^{2^{n}-1}\;P_{m}F_{m}Ex\left(\xi -1;x\left(0\right)\right)=G_{1}\delta_{2^{n}}^{\chi }=\delta_{2^{n}}^{\chi }. \end{array}$$
- if *ξ* = *t*_*k*_, one has
  $$\begin{array}{c} Ex\left(\xi ;x\left(0\right)\right)=E\cdot Ex\left(\xi -1;x\left(0\right)\right)=G_{2}\delta_{2^{n}}^{\chi }=\delta_{2^{n}}^{\chi }. \end{array}$$

Therefore, there exists a positive integer *k* such that

$$\begin{array}{c} P_{r}\{x\left(t;x\left(0\right)\right)=\delta_{2^{n}}^{\chi }\}=1 \end{array}$$

holds for any integer *t* ≥ *k* and any *x*(0) ∈ Δ_2^*n*^_. By Definition 4, GAPBN with impulsive effects (1) is finite-time stable at *x*_*d*_ with probability one.

(Necessity): Suppose that GAPBN with impulsive effects (1) is finite-time stable at $\delta_{2^{n}}^{\chi }$ with probability one. Then, from Definition 4, there exists a positive integer *k* such that

$$\begin{array}{c} P_{r}\{x\left(k;x\left(0\right)\right)=\delta_{2^{n}}^{\chi }\}=1 \end{array}$$

holds for any initial state *x*(0) ∈ Δ_2^*n*^_. Hence,

$$\begin{array}{c} Ex\left(k;x\left(0\right)\right)=\delta_{2^{n}}^{\chi }. \end{array}$$

From (8), one has ${\tilde{F}}_{k}x\left(0\right)=\delta_{2^{n}}^{\chi }$, which along with the arbitrariness of *x*(0) shows that $Row_{d}\{{\tilde{F}}_{k}\}=1_{2^{n}}^{T}.$

Based on Definition 3 and Definition 5, one has the following corollary.

**Corollary 2.** The GAPBN with impulsive effects (1) achieves inner synchronization, if and only if it can be stabilized into the synchronization set $\Omega =\{\delta_{2^{n}}^{1},\delta_{2^{n}}^{2^{n}}\}.$

**Proof.** (Sufficiency): Assuming the system set stabilizes into set $\Omega =\{\delta_{2^{n}}^{1},\delta_{2^{n}}^{2^{n}}\}$, then the system state *x*(*t*) will only transition between $\delta_{2^{n}}^{1}$ and $\delta_{2^{n}}^{2^{n}}$, where *t* ≥ *k*. Each node state can only be $\delta_{2}^{1}$ or $\delta_{2}^{2}$, then one has $P_{r}\{x_{1}\left(t\right)=x_{2}\left(t\right)=\ldots =x_{n}\left(t\right)=\delta_{2}^{1}\left(\delta_{2}^{2}\right)\}=1$, thus the GAPBN with impulsive effects (1) is synchronized.

(Necessity): Suppose that GAPBN with impulsive effects (1) is synchronized. Then we have *P*_*r*_ {*x*_1_(*t*) = $\ldots =x_{n}\left(t\right)=\delta_{2}^{1}\left(\delta_{2}^{2}\right)$} = 1, where *t* ≥ *k*. Thus, *P*_*r*_{*x*(*t*; *x*(0)) ∈ Ω} = 1, $\Omega =\{\delta_{2^{n}}^{1},\delta_{2^{n}}^{2^{n}}\}$. Hence, the system stabilizes into set Ω.

## Examples

### Set stability

Consider the following GAPBN with impulsive effects:

$$\begin{array}{c} \left\{ \begin{array}{c} x_{1}\left(t+1\right)=x_{1}\left(t\right)\vee x_{3}\left(t\right),\\ x_{2}\left(t+1\right)=\neg x_{2}\left(t\right)\to x_{3}\left(t\right),\\ x_{3}\left(t+1\right)=x_{1}\left(t\right)\wedge x_{2}\left(t\right). \end{array}\right. \end{array}$$ (10)

At the moment of the impulse (*t* = *t*_*k*_ − 1), there is the following BN.

$$\begin{array}{c} \left\{ \begin{array}{c} x_{1}\left(t+1\right)=x_{2}\left(t\right)\vee x_{3}\left(t\right),\\ x_{2}\left(t+1\right)=x_{1}\left(t\right)\wedge x_{3}\left(t\right),\\ x_{3}\left(t+1\right)=x_{2}\left(t\right), \end{array}\right. \end{array}$$ (11)

where *t*_*k*_ = *k*^2^ + 1, $k\in Z_{+}$, the probability of updating at each node is *p*_1,1_ = 0.8, *p*_2,1_ = 0.4 and *p*_3,1_ = 0.5 respectively.

For a GAPBN with three nodes, there are 2^3^ = 8 different update situations at time *t*.

(1) When all nodes are not updated, *x*(*t* + 1) = *x*_1_(*t*)*x*_2_(*t*)*x*_3_(*t*) = *I*_8_*x*(*t*) = *δ*_8_[1 2 3 4 5 6 7 8]*x*(*t*) = *F*_0_*x*(*t*) and *P*_0_ = 0.06;
(2) When only the *x*_1_ is updated, *x*(*t* + 1) = *x*_1_(*t* + 1)*x*_2_(*t*)*x*_3_(*t*) = *δ*_8_[1 2 3 4 1 6 3 8]*x*(*t*) = *F*_1_*x*(*t*) and *P*_1_ = 0.24;
(3) When only the *x*_2_ is updated, *x*(*t* + 1) = *x*_1_(*t*)*x*_2_(*t* + 1)*x*_3_(*t*) = *δ*_8_[1 2 1 4 5 6 5 8]*x*(*t*) = *F*_2_*x*(*t*) and *P*_2_ = 0.06;
(4) When only the *x*_3_ is updated, *x*(*t* + 1) = *x*_1_(*t*)*x*_2_(*t*)*x*_3_(*t* + 1) = *δ*_8_[1 1 4 4 6 6 8 8]*x*(*t*) = *F*_3_*x*(*t*) and *P*_3_ = 0.06;
(5) When only the *x*_1_, *x*_2_ are updated, *x*(*t* + 1) = *x*_1_(*t* + 1)*x*_2_(*t* + 1)*x*_3_(*t*) = *δ*_8_[1 2 1 4 1 6 1 8]*x*(*t*) = *F*_4_*x*(*t*) and *P*_4_ = 0.16;
(6) When only the *x*_1_, *x*_3_ are updated, *x*(*t* + 1) = *x*_1_(*t*+ 1)*x*_2_(*t*)*x*_3_(*t* + 1) = *δ*_8_[1 1 4 4 2 6 4 8]*x*(*t*) = *F*_5_*x*(*t*) and *P*_5_ = 0.24;
(7) When only the *x*_2_, *x*_3_ are updated, *x*(*t* + 1) = *x*_1_(*t*)*x*_2_(*t* + 1)*x*_3_(*t* + 1) = *δ*_8_[1 1 2 4 6 6 6 8]*x*(*t*) = *F*_6_*x*(*t*) and *P*_6_ = 0.04;
(8) When only the *x*_1_, *x*_2_, *x*_3_ are updated, *x*(*t* + 1) = *x*_1_(*t* + 1)*x*_2_(*t* + 1)*x*_3_(*t* + 1) = *δ*_8_[1 1 2 4 2 6 2 8]*x*(*t*) = *F*_7_*x*(*t*) and *P*_7_ = 0.16.

**Fig 1:**
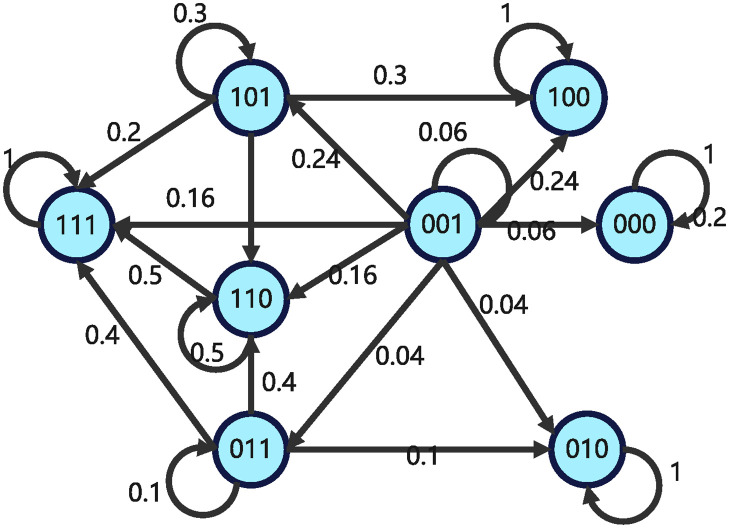
State transition diagram of GAPBN with impulsive effects at non pulse time.

**Fig 2:**
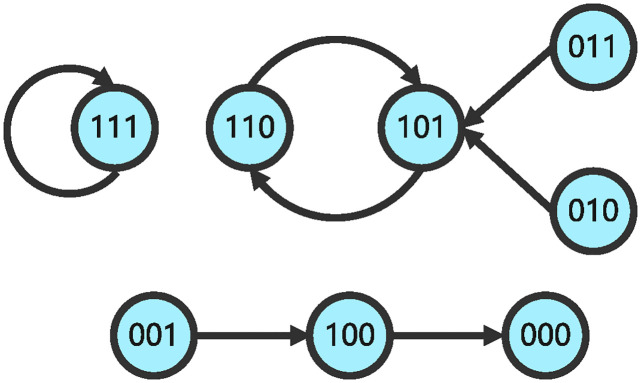
State transition diagram of GAPBN with impulsive effects at pulse time.

The structure matrix of impulsive influence is *E* = *δ*_8_[1 3 2 8 3 3 4 8]. The state transition relationships of GAPBN (10) and impulsive effects (11) are shown in Figs 1 and 2. Next, our objective is to check whether system is finite-time stable at $\Omega =\{\delta_{8}^{1},\delta_{8}^{2},\delta_{8}^{4},\delta_{8}^{8}\}$ with probability one. Through Algorithm 1, one can get $I\left(\Omega \right)=\{\delta_{8}^{1},\delta_{8}^{4},\delta_{8}^{8}\}$.

Let *F* = [*F*_0_, *F*_1_, *F*_2_, *F*_3_, *F*_4_, *F*_5_, *F*_6_, *F*_7_] = *δ*_8_[1 2 3 4 5 6 7 8 1 2 3 4 1 6 3 8 1 2 1 4 5 6 5 8 1 1 4 4 6 6 8 8 1 2 1 4 1 6 1 8 1 1 4 4 2 6 4 8 1 1 2 4 6 6 6 8], $P={\left[P_{0},P_{1},P_{2},P_{3},P_{4},P_{5},P_{6},P_{7}\right]}^{T}=[0.06\;0.24\;0.04\;0.06\;0.16\;0.24\;0.04\;0.16{]}^{T}$, then

$$\begin{array}{c} G_{1}=F⋉P=[\begin{array}{cccccccc} 1 & 0.5 & 0.2 & 0 & 0.4 & 0 & 0.16 & 0\\ 0 & 0.5 & 0.2 & 0 & 0.4 & 0 & 0.16 & 0\\ 0 & 0 & 0.3 & 0 & 0 & 0 & 0.24 & 0\\ 0 & 0 & 0.3 & 1 & 0 & 0 & 0.24 & 0\\ 0 & 0 & 0 & 0 & 0.1 & 0 & 0.04 & 0\\ 0 & 0 & 0 & 0 & 0.1 & 1 & 0.04 & 0\\ 0 & 0 & 0 & 0 & 0 & 0 & 0.06 & 0\\ 0 & 0 & 0 & 0 & 0 & 0 & 0.06 & 1 \end{array}], \end{array}$$

and *G*_2_ = *E* = *δ*_8_[1 3 2 8 3 3 4 8].

Based on (5), one can see that

$$\begin{array}{c} \sum_{j\in I(\Omega )}\;Row_{j}\left({\hat{F}}_{20}\right)=\left[1,1,1,1,1,1,1,1\right]. \end{array}$$

From Theorem 2, system (10) and syetem(11) are finite-time Ω-stable with prbability one.

### Synchronization

Consider the following GAPBN with impulsive effects:

$$\begin{array}{c} \left\{ \begin{array}{c} x_{1}\left(t+1\right)=x_{1}\left(t\right)\vee x_{2}\left(t\right),\\ x_{2}\left(t+1\right)=x_{1}\left(t\right). \end{array}\right. \end{array}$$ (12)

At the moment of the impulse (*t* = *t*_*k*_ − 1), there is the following BN.

$$\begin{array}{c} \left\{ \begin{array}{c} x_{1}\left(t+1\right)=x_{1}\left(t\right)\to x_{2}\left(t\right),\\ x_{2}\left(t+1\right)=x_{1}\left(t\right)↔x_{2}\left(t\right). \end{array}\right. \end{array}$$ (13)

where *t*_*k*_ = *k*^2^ + 2, $k\in Z_{+}$, the probability of updating at each node is *p*_1,1_ = 0.5 and *p*_2,1_ = 0.5 respectively.

**Fig 3:**
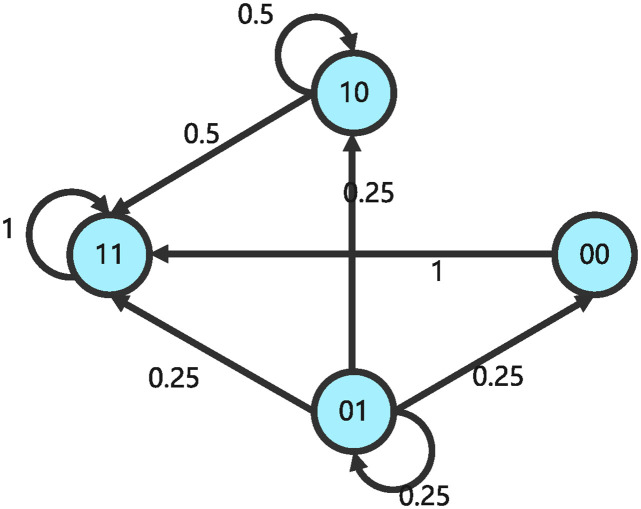
State transition diagram of GAPBN with impulsive effects at non pulse time.

**Fig 4:**
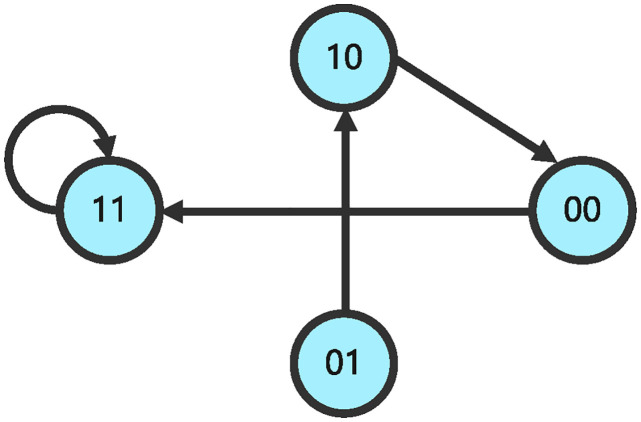
State transition diagram of GAPBN with impulsive effects at pulse time.

For a GAPBN with two nodes, there are 2^2^ = 4 different update situations, and the corresponding structure matrices and probabilities are

$$\begin{array}{c} \left\{ \begin{array}{c} F_{0}=\delta_{4}\left[1\;2\;3\;4\right],\;P_{0}=0.25,\\ F_{1}=\delta_{4}\left[1\;12\;4\right],\;P_{1}=0.25,\\ F_{2}=\delta_{4}\left[1\;1\;4\;4\right],\;P_{2}=0.25,\\ F_{3}=\delta_{4}\left[1\;2\;1\;4\right],\;P_{3}=0.25, \end{array}\right. \end{array}$$

and the structure matrix of impulsive influence is *E* = *δ*_4_ [1 4 2 1]. The state transition relationships of GAPBN (12) and impulsive effects (13) are shown in Figs 3 and 4.

Our objective is to check if the system is synchronized, i.e., whether the system is finite-time stable at $\Omega =\{\delta_{4}^{1},\delta_{4}^{4}\}$ with probability one. Through Algorithm 1, one can get $I\left(\Omega \right)=\{\delta_{4}^{1},\delta_{4}^{4}\}$.

Let *F* = [*F*_0_, *F*_1_, *F*_2_, *F*_3_] = *δ*_4_[1 2 3 4 1 1 2 4 1 1 4 4 1 2 1 4], $P={\left[P_{0},P_{1},P_{2},P_{3}\right]}^{T}=[0.25\;0.25\;0.25\;0.25{]}^{T}$, then

$$\begin{array}{c} G_{1}=F⋉P=[\begin{array}{cccc} 1 & 0.5 & 0.25 & 1\\ 0 & 0.5 & 0.25 & 0\\ 0 & 0 & 0.25 & 0\\ 0 & 0 & 0.25 & 0 \end{array}],G_{2}=E=[\begin{array}{cccc} 1 & 0 & 0 & 1\\ 0 & 0 & 1 & 0\\ 0 & 0 & 0 & 0\\ 0 & 1 & 0 & 0 \end{array}]. \end{array}$$

Based on (5), one can see that

$$\begin{array}{c} \sum_{j\in I(\Omega )}\;Row_{j}({\hat{F}}_{3})=\left[1,1,1,1\right]. \end{array}$$

From Theorem 2, system (12) and system (13) are finite-time Ω-stable with probability one. According to Corollary 2, one can get that the system (12) and system (13) have reached synchronization.

## Conclusion

In this study, GAPBNs with impulsive effects, as one kind of discrete dynamic system, were investigated on set stability. Firstly, based on the theory of STP, we expressed the state space of GAPBNs with impulsive effects in algebraic form. Secondly, an algorithm was designed to decide the largest invariant set of the system. Due to the influence of impulses, the system state may deviate from the largest invariant set of the initial network. Therefore, it is essential to shrink the largest invariant set to ensure the stability of the system. After that, based on the shrunk largest invariant set, a sufficient and necessary condition for the set stability of GAPBNs with impulsive effects is proposed. In the end, according to the different sets, corollaries about global stability and synchronization are given. In the future, we will integrate homogeneous Markov chain into GAPBNs, with purpose of investigating the set stability of GAPBNs. The other future work is to further analyze the external synchronization criteria of GAPBNs with impulsive effects.
